# Identification of Reference Genes for RT-qPCR Assays in *Sambucus nigra* L.

**DOI:** 10.3390/cimb48080776

**Published:** 2026-07-30

**Authors:** Zhengkun Cui, Qian Zhang, Shengyu Gao, Yinyin Fu, Yin Sun, Fei Ren, Junxiu Yao

**Affiliations:** 1 Shandong Academy of Forestry, Jinan 250014, China; zkcui@njfu.edu.cn (Z.C.); sdslkyzq@163.com (Q.Z.); fuyinyin1029@163.com (Y.F.); sunyin156256156256@163.com (Y.S.); lkyrenfei@163.com (F.R.); 2 College of Food Science and Engineering, Shandong Agriculture and Engineering University, Jinan 255300, China; gaoshengyu73@163.com

**Keywords:** *Sambucus nigra* L., reference genes, gene expression stability

## Abstract

Reverse transcription quantitative PCR (RT-qPCR) is one of the most widely used techniques for gene expression analysis in molecular biology. However, the accuracy of relative gene expression quantification largely depends on the stability of the reference genes used for normalization. *Sambucus nigra* L. (elderberry) is a valuable medicinal and edible plant rich in anthocyanins and other bioactive compounds. Despite its increasing research and application value, no reference genes have been validated for this species. In this study, we applied RT-qPCR alongside four algorithms (GeNorm, NormFinder, BestKeeper, and RefFinder) to assess the expression stability of nine candidate reference genes across ten samples representing five tissue types (including stems, flowers, leaves, roots, fruits) at different developmental stages. The results showed that *VAMP* and *Pol* were the most suitable reference gene combination for normalization across different tissues of *S. nigra*. For studies involving only vegetative tissues (leaves and stems), our results recommend that *RPB2* and *RPB5* are the most stable and suitable reference genes. This study provides reliable reference genes for accurate RT-qPCR-based gene expression analysis in *S. nigra* and establishes a useful methodological basis for future functional genomics and molecular breeding studies in this species.

## 1. Introduction

Reverse transcription quantitative PCR (RT-qPCR) is a sensitive, specific, and widely used technique for quantitative analysis of gene expression. It has been extensively applied in molecular biology, functional genomics, and gene regulatory studies [1,2]. By monitoring fluorescence signals during amplification, RT-qPCR enables the quantitative detection of target gene transcript abundance [2]. However, the reliability of RT-qPCR results depends strongly on the selection of appropriate reference genes for data normalization [3]. Therefore, identifying stable reference genes is essential for reducing biological variation and technical bias among different samples [4,5,6].

The validation of suitable reference genes is a critical prerequisite for accurate RT-qPCR analysis [7]. An ideal reference gene should maintain stable expression across different tissues, developmental stages, experimental treatments and sample qualities [8,9,10]. However, the expression stability of commonly used reference genes is not universal. It can vary substantially among species, tissue types and experimental conditions [11]. Therefore, reference genes should be systematically evaluated before being used in a specific species or experimental system [12].

Computational algorithms have themselves been proven reliable for reference gene screening across multiple species, including trees, shrubs, and herbaceous plants, such as *Rehmannia* spp. [13], blueberry [14] and *Litsea coreana* [15]. These studies usually assess the expression stability of candidate reference genes using multiple statistical algorithms, followed by an integrated ranking to identify the most reliable genes for normalization. The most commonly used tools include geNorm, NormFinder, BestKeeper, and RefFinder [16]. GeNorm calculates expression stability (M values) and pairwise variation (V values) [4]. NormFinder and BestKeeper assess stability using statistical parameters such as standard deviation (SD) and coefficient of variation (CV) from raw cycle threshold (Ct) values [17,18]. RefFinder generates candidate rankings via geometric averaging of these data [19].

*Sambucus nigra* L., commonly known as elderberry, is a deciduous shrub with strong ecological adaptability and broad cultivation potential [20,21]. Its natural distribution extends from the Tatra Mountains in Poland in the north to the Atlas Mountains in North Africa in the south, and from the Iranian Plateau in the east to the western coastal regions of Scotland [22,23]. At present, *S. nigra* has also been introduced to many regions worldwide, including East Asia and North America, reflecting its broad adaptability [24]. The increasing interest in elderberry is mainly driven by its high economic and medicinal value. Elderberry fruits are rich in phenolic compounds, especially anthocyanins, which contribute to strong antioxidant activity. For example, the cultivar ‘Sampo’ can accumulate anthocyanins up to 1800 mg per 100 g fresh weight [25]. In addition, fruit and flower extracts of elderberry have been reported to relieve symptoms of respiratory infections [26,27]. Elderberry flower extracts have also been recommended by the German Commission and the European Medicines Agency (EMA) for the treatment of upper respiratory tract infections [28,29]. Given the importance of its bioactive metabolites, accurate gene expression analysis is needed to investigate key biosynthetic and regulatory pathways in *S. nigra*. However, the lack of validated reference genes currently limits reliable RT-qPCR-based expression analysis in this species.

In this study, we evaluated the expression stability of nine candidate reference genes in *S. nigra*, including *ACTIN*, *CYP* (*Cyclophilin*), *RPL* (*50S ribosomal protein*), *UBC* (*Ubiquitin-conjugating enzyme*), *RPB* (*RNA polymerase II subunit*), *D6PK* (*D6 protein kinase*), *Pol* (*RNA polymerase subunit 12*) and *VAMP* (*Vesicle-associated membrane protein*). Samples from multiple tissues, including roots, stems, leaves, flowers and fruits, were used for stability assessment. Four widely used algorithms, geNorm, NormFinder, BestKeeper and RefFinder, were applied to compare candidate gene stability. By integrating the results from these methods, we identified stable reference genes suitable for different tissue groups in *S. nigra*. This work provides a practical reference gene set for accurate gene expression normalization and supports future molecular studies in elderberry.

## 2. Materials and Methods

### 2.1. Plant Materials

*S. nigra* plant materials were collected from Feixian County State-owned Benghe Forest Farm, Shandong Province (E 118°12′, N 35°22′). A total of five tissues were collected, including leaf, flower, fruit, stem, and root (Table 1). Leaf samples were harvested at 3 stages: Stage 1 represented leaf buds, Stage 2 corresponded to newly expanded leaves, and Stage 3 leaf samples were mature leaves. Flower samples were harvested at 2 stages: Stage 1 samples were collected in the full bloom period, whereas Stage 2 samples were at the period when the ovary had developed into fruit. Fruit samples included two developmental stages: Stage 1 was immature green fruits, and Stage 2 was mature black fruits. Stem samples were collected at two distinct parts: Stage 1 was current-year newly sprouted shoots, and Stage 2 was perennial shoots on the main stems. Root samples were collected from excavated one-year-old potted *S. nigra*. Owing to the high lignification of taproots, which posed challenges for RNA extraction, lateral roots were selected as representative root samples. Each tissue was collected from three independent plants (*n* = 3). Samples were flash-frozen in liquid nitrogen and stored at −80 °C for RNA extraction.

**Table 1:** Test plant material samples.

Sample		Sampling Time	The Code in Figure 1
Leaf	Stage 1	16 May 2024	A1
	Stage 2	16 May 2024	A2
	Stage 3	16 May 2024	A3
Flower	Stage 1	16 May 2024	B1
	Stage 2	6 June 2024	B2
Fruit	Stage 1	4 July 2024	C1
	Stage 2	28 July 2024	C2
Stem	Stage 1	16 May 2024	D1
	Stage 2	16 May 2024	D2
Root	lateral root	15 July 2024	E

### 2.2. Total RNA Extraction and cDNA Synthesis

Total RNA was extracted using the RNAprep Pure Plant Kit (Tiangen, Beijing, China) per the manufacturer’s protocol. RNA concentration and quality were evaluated via NanoDrop 2000c spectrophotometer (Thermo Scientific, Wilmington, DE, USA) and 1.0% (*w*/*v*) agarose gel electrophoresis. First-strand cDNA synthesis was performed using 500 ng total RNA using the PrimeScript^TM^ RT Master Mix (TaKaRa, Dalian, China) in 20 μL reactions, followed by dilution to 200 μL with nuclease-free water. cDNA extracted from S1 leaves was serially diluted (10-fold, 100-fold, and 1000-fold) and used as a standard for constructing the calibration curve. A standard curve was generated by plotting the logarithm of the standard concentration (log_10_ copy number) on the *X*-axis against the corresponding threshold cycle (Ct) values on the *Y*-axis. Linear regression analysis yielded the equation of the standard curve: Ct = k × log_10_(concentration) + b. The amplification efficiency (E) was calculated from the slope of the standard curve using the formula: E = 10^(–1/slope)^ − 1. The coefficient of determination (R^2^), obtained through linear regression analysis, was used to evaluate the goodness of fit between the data points and the regression line in the standard curve.

### 2.3. Candidate Reference Genes Selection and Primer Design

Candidate reference genes were selected in this study based on transcriptomic sequencing data from *S. nigra* tissues. Transcript expression levels were quantified using RNA-Seq by Expectation-Maximization (RSEM), with Fragments Per Kilobase of exon model per Million mapped fragments (FPKM) measuring expression abundance. Transcripts with FPKM < 50 in any tissue were filtered out. This step was performed to filter out transcripts with low expression levels. Remaining transcripts underwent further screening. The longest open reading frame (ORF) transcript was selected to represent the coding DNA sequence (CDS). These CDS FPKM across tissues were scored by standard deviation. Based on ranking by standard deviation, the top-ranked sequences became candidate sequences. Finally, we utilized the NCBI database to identify these candidate reference genes. This step was to identify stable expression profiles in *S. nigra*. Ultimately, 3 candidate reference genes were identified.

Further, hidden Markov models (HMMs) of common plant reference genes (e.g., *ACTIN*, *UBC*, *RPL*) were downloaded from Pfam (http://pfam.xfam.org/, accessed on 14 August 2024) [30]. These genes have been identified as suitable for reference genes in previous studies [31,32]. These HMMs were aligned to the CDS file using HMMER 3.0 (http://hmmer.org/, default E-values). Finally, 12 candidate genes were identified from the HMMs: 1 *ACTIN* gene, 2 *CYP* genes, 2 *RPL* genes, 3 *UBC* genes, 4 *RPB* genes. 3 candidate genes were identified from standard deviation rank: *D6PK*, *Pol*, and *VAMP*. Their RT-qPCR primers were designed using the Oligo 7 algorithm (Molecular Biology Insights, Inc., Cascade, CO, USA, https://en.freedownloadmanager.org/Windows-PC/OLIGO.html, accessed on 14 August 2024) and renamed based on gene family classification (Table 2). *RPB4* and *UBC2* were not detected during HMM screening and are thus absent from the candidate gene list. Design parameters were as follows: primer melting temperature (Tm) 58–63 °C, primer length 18–22 bp, GC content 40–60%, and amplicon length 90–200 bp. The amplification efficiencies were predicted using the LinRegPCR 2014.x algorithm (Amsterdam University, Amsterdam, the Netherlands, http://LinRegPCR.nl, accessed on 14 August 2024) to estimate the mean amplification efficiencies of the primer pairs [33]. Primer specificity was validated via 2.0% (*w*/*v*) agarose gel electrophoresis and melting curve analysis.

### 2.4. Fluorescence Quantitative PCR Amplification of Candidate Reference Genes

Quantitative analysis was performed following the manufacturer’s protocol. Extracted plant tissue RNA was reverse-transcribed into cDNA for subsequent experiments. Each reaction had a total volume of 10 μL. The 10 μL reaction mixture comprised: 5 ng cDNA (1 μL), 10 pmol each of forward/reverse primers, 5 μL 2× SYBR Ex Taq Premix, 0.2 μL 50× ROX Reference Dye (TaKaRa, Dalian, China), with the remaining volume adjusted using deionized water.

The amplification program was: 95 °C for 30 s; 40 cycles of 95 °C for 5 s and 72 °C for 20 s. After amplification, melting curve analysis verified primer specificity via a dissociation protocol: 95 °C for 15 s, 60 °C for 1 min, 95 °C for 15 s. All RT-qPCR reactions included three biological and three technical replicates. Amplification efficiency ranged from 85% to 115%, with correlation coefficients exceeding 0.98, meeting experimental standards [34].

### 2.5. Data Analysis

The expression stability of the candidate genes was evaluated by four algorithms, GeNorm, NormFinder, BestKeeper, and RefFinder. The optimal reference genes were selected based on systematic integration of four-program ranking averages. We converted relative expression to the cycle threshold (Ct) values. We used the formula E^−∆Ct^, where ∆Ct is the difference between the Ct value of each gene and the minimum Ct value, and E is the mean amplification efficiency of the corresponding primer pair. These data were used for GeNorm and NormFinder operation.

The GeNorm algorithm is commonly used to evaluate gene expression stability. It uses the above data to generate the average expression stability value (M) for evaluating candidate primer pairs, and uses paired variation (V_n/n+1_) to determine the optimal number of reference genes. The average paired variation between a specific candidate gene and all other candidate reference genes is called the M-value, and paired variation (V_n/n+1_) is the standard deviation of the logarithmic transformation expression rate of the combination of two candidate reference genes [4].

The NormFinder algorithm is a statistical tool that calculates the intragroup and intergroup expression variation in candidate reference genes. It evaluated our candidate primer pairs’ expression stability and ranked them to screen for the most suitable reference genes for RT-qPCR [17].

The BestKeeper algorithm is an Excel-based tool that analyzes raw Ct values without conversion, calculates the SD, CV, and Pearson correlation coefficient (r) between candidate reference genes and other genes to evaluate their expression stability [18].

Ultimately, the RefFinder online algorithm (http://blooge.cn/RefFinder/, accessed on 20 December 2024) was used for the final ranking. It integrated the results of geNorm, NormFinder, BestKeeper, and Δ Ct methods, and generated the final stability ranking by calculating the geometric mean of gene rankings. It produced a final stability score, thereby avoiding the limitations of any single algorithm. This approach made it suitable for expression normalization studies under diverse tissue-specific experimental conditions [19].

### 2.6. Reference Gene Stability Verification

The candidate genes with the most stable and unstable expression levels were selected. *SnAHCY* was selected as the target gene. We chose leaf (s1, s3), stem (s1), flower (s1), fruit (s1), and root as the test samples because the expression levels of *SnAHCY* showed significant differences among these samples. The primer pairs (forward:5′-TCCCACCTCAACTGACAACG-3′ and reverse: 5′-ACACCGACCAACCTCTCCTT-3′, Amplification efficiency (E): 107.1694% and Correlation coefficient (R^2^): 99.61%) were used for RT-qPCR. Their expression was calculated by the formula 2^−∆∆CT^.

## 3. Results

### 3.1. Primer Specificity Evaluation of Reference Gene

**Figure A1:**
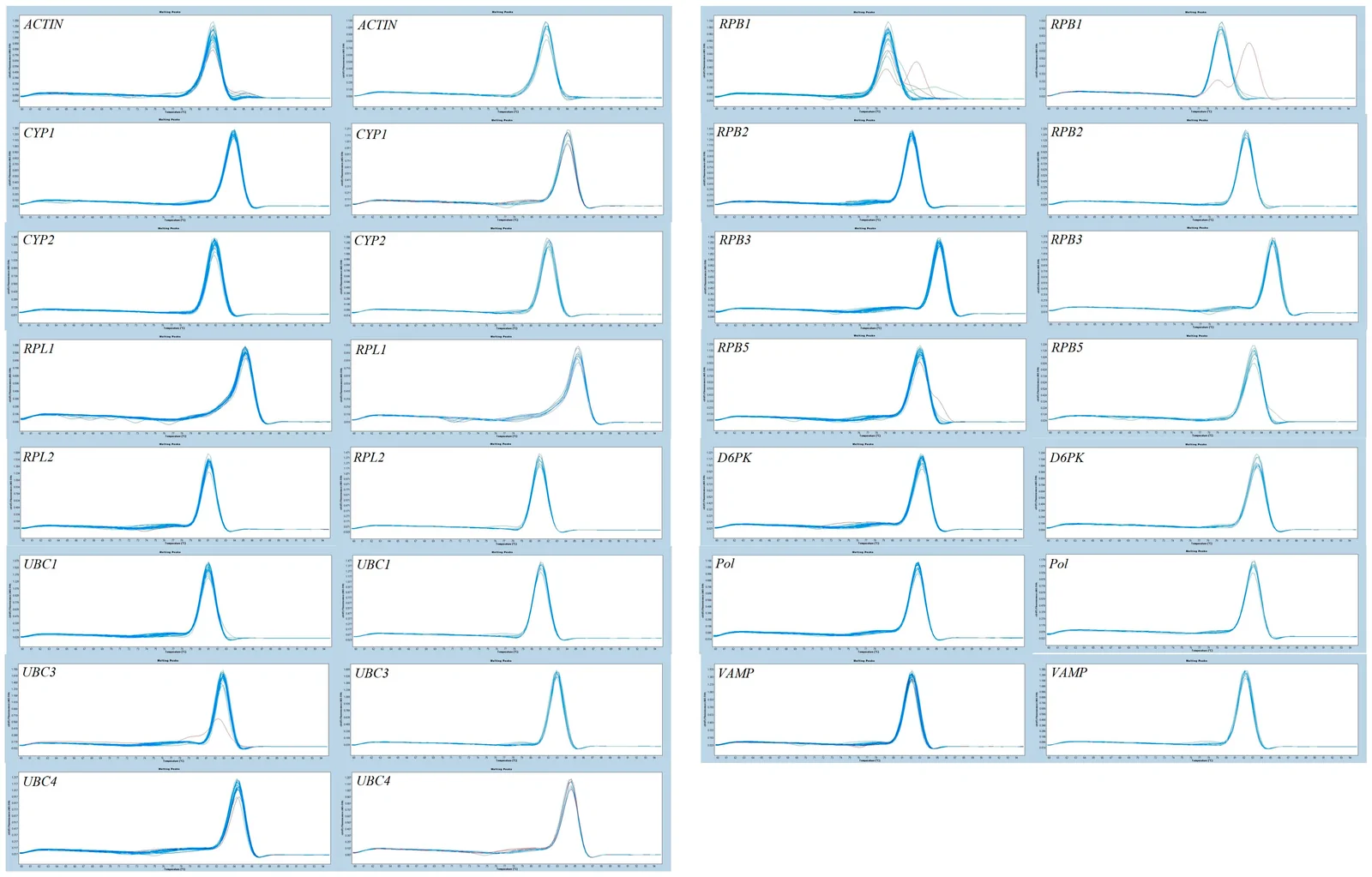
The melting curve of 15 candidate reference genes. Each gene corresponds to two images, with the left representing the vegetative organs and the right representing the reproductive organs. A single, sharp peak indicates specific amplification of the target gene. All genes except *RPL1* showed a single defined melting peak, demonstrating specific product formation. The *RPL1* sample displayed an additional small shoulder peak, suggesting the presence of non-specific amplification products.

A normal primer melting curve indicates specific amplification with a single distinct peak, confirming the absence of primer dimers or non-specific products, ensuring reliable RT-qPCR results. We avoid the occurrence of hairpin structures and primer dimers in primer design. However, the RT-qPCR dissolution curve of *RPB1* exhibited a clear dual-melting-peak phenomenon, and it was excluded from the candidate reference primers. No abnormalities were observed in the other candidate 14 genes (Figure A1).

Using cDNA derived from s1 leaves at different dilution ratios (undiluted, 10-fold, 100-fold, and 1000-fold), we evaluated the amplification efficiency of the remaining primers. Standard curves were generated for each primer pair, from which the real-time amplification efficiency and correlation coefficient (R^2^) were determined.

RT-qPCR parameters are detailed in Table 3. Nine genes (*RPL1*, *RPL2*, *UBC4*, *RPB2*, *RPB3*, *RPB5*, *D6PK*, *Pol*, *VAMP*) demonstrated amplification parameters: 85–115% efficiency with correlation coefficients > 0.98, conforming to RT-qPCR standards. These nine genes were therefore selected for subsequent reference gene evaluation.

### 3.2. Analysis of Reference Gene Expression Profile

**Figure 2:**
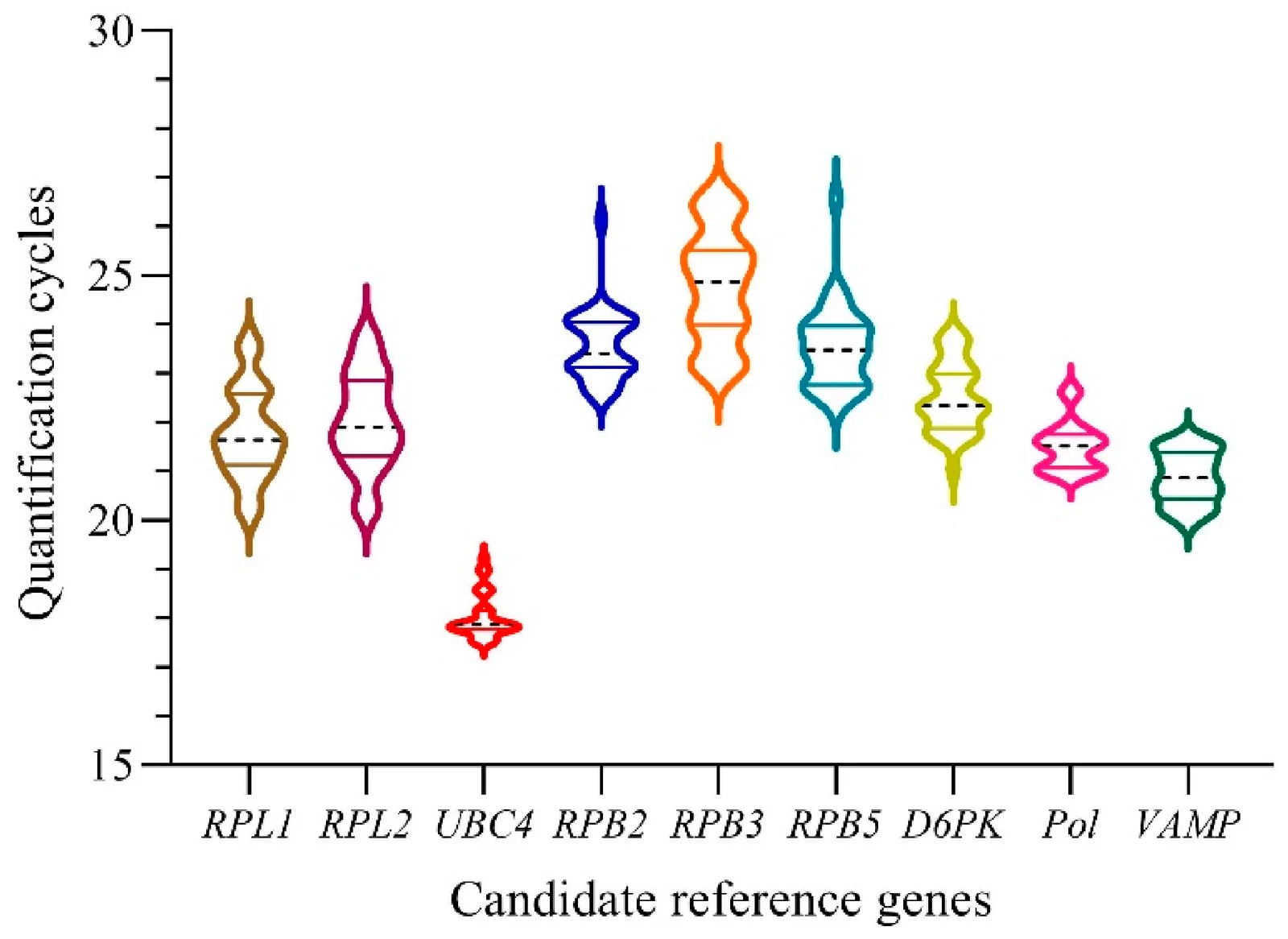
Violin plot of Ct values for 9 candidate reference genes in *S. nigra*. The width of the violin plot represents the kernel density distribution of Ct values, where wider sections indicate a higher frequency of samples with that Ct value. Colored horizontal lines denote the 25th and 75th percentiles, and black dashed lines represent the median Ct values.

The Ct values of nine candidate reference genes across ten samples are shown in Figure 2, with an overall range of 17.49 to 26.59. *UBC4* exhibited the smallest variation (Ct range: 17.49–19.22), indicating the most stable expression. It also showed the lowest intragroup maximum and minimum values. *Pol* ranked second in stability (20.90–22.69), followed by *VAMP* (19.91–21.73). In contrast, *RPB5* displayed the largest variation (21.04–26.59) and the highest intragroup maximum value. *RPL2* (20.24–23.84) and *RPB2* (22.55–26.14) ranked second and third in variability, respectively.

### 3.3. Stability Analysis of Reference Genes in Different Tissues

#### 3.3.1. Analysis of geNorm

**Figure 3:**
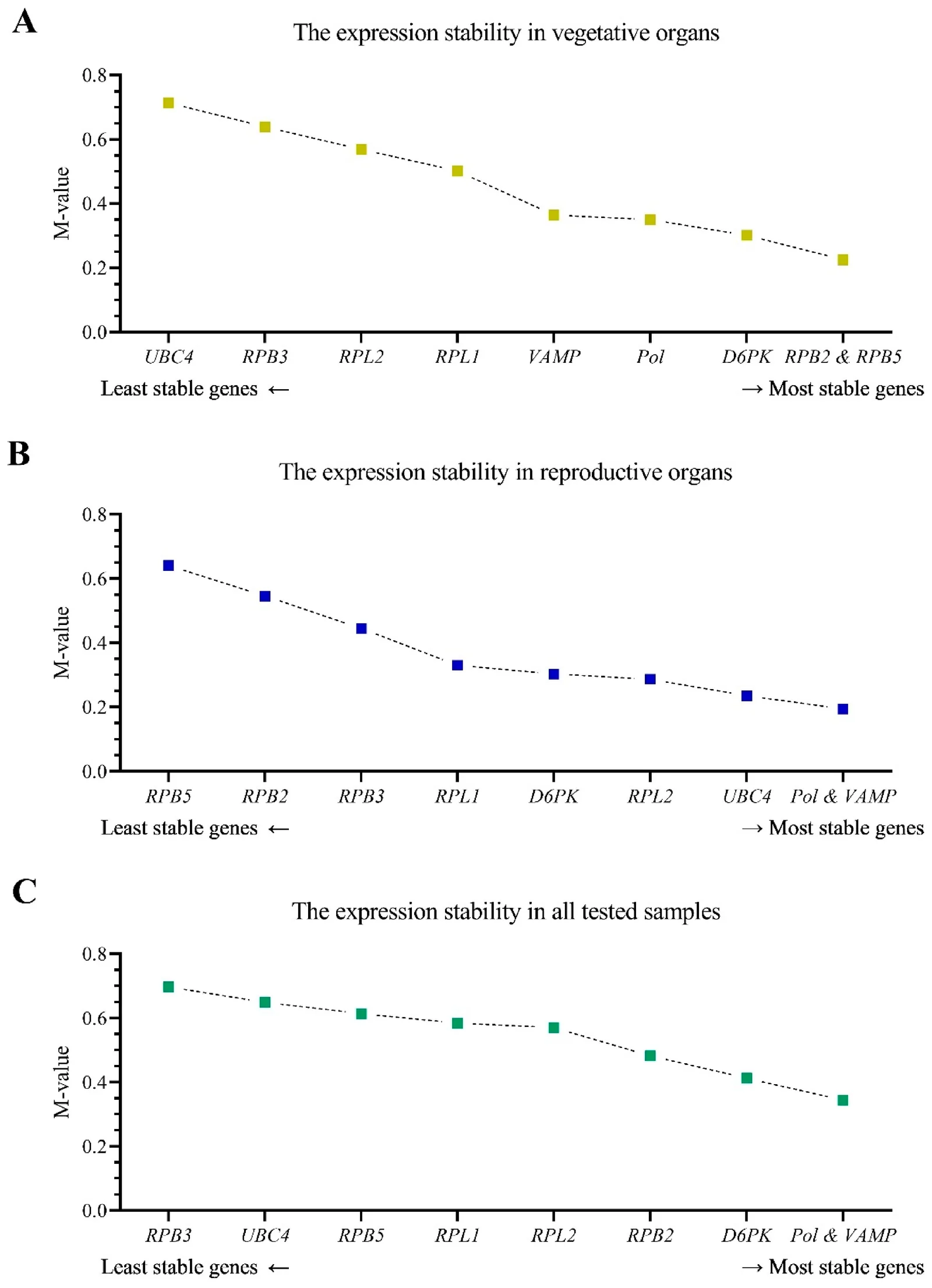
Average expression stability of 9 candidate reference genes analyzed by GeNorm. (**A**) vegetative organs; (**B**) reproductive organs; (**C**) all tested samples. The *x*-axis represents the rank, and the *y*-axis indicates the M-value. Yellow, blue, and green dots correspond to expression stability in vegetative organs, reproductive organs, and all samples.

The geNorm software (version 3.4) evaluated the expression stability of nine candidate reference genes in samples by M-value. A candidate reference gene with an M-value higher than 1.5 is considered unstable, and a higher M-value indicates lower expression stability [4]. The candidate reference genes exhibited M-values below 1.5 across all sample groups, including vegetative organs (stems and leaves, Figure 3A), reproductive organs (flowers and fruits, Figure 3B), and the complete sample set (Figure 3C). According to the geNorm algorithm, *RPB2* and *RPB5* were the most stable reference genes in vegetative organs, with an M-value of 0.224. In contrast, these two genes exhibited the lowest stability in reproductive organs, with M-values of 0.546 and 0.641, respectively. The most stable reference genes in reproductive tissues were *Pol* and *VAMP*, with a co-ranked M-value of 0.194. This gene pair also showed the best overall performance across all samples, with a co-ranked M-value of 0.344. *UBC4* performed well in reproductive organs (M-value = 0.234) but was the least stable in vegetative organs and ranked second worst in the complete sample set. *RPB3* consistently ranked among the bottom three across all 3 sample groups.

It is worth noting that *UBC4* exhibited the narrowest raw Ct range (17.49–19.22), yet was ranked as the least stable gene in vegetative organs and second-worst in the complete sample set by GeNorm analysis. This apparent discrepancy arises because the raw Ct range and the GeNorm M-value measure different aspects of the data. The raw Ct range reflects only the absolute spread of Ct values for a single gene, whereas the GeNorm M-value is calculated from the pairwise variation in relative expression ratios between all candidate genes. *UBC4*’s exceptionally low Ct values relative to other genes (e.g., *RPB5*: 21–27) caused even small technical fluctuations in its Ct to be amplified when computing expression ratios with other genes, resulting in higher pairwise variability and a poorer M-value. This highlights the importance of using multiple complementary algorithms for reference gene selection, as a gene may perform well on one metric but poorly on another.

**Figure 4:**
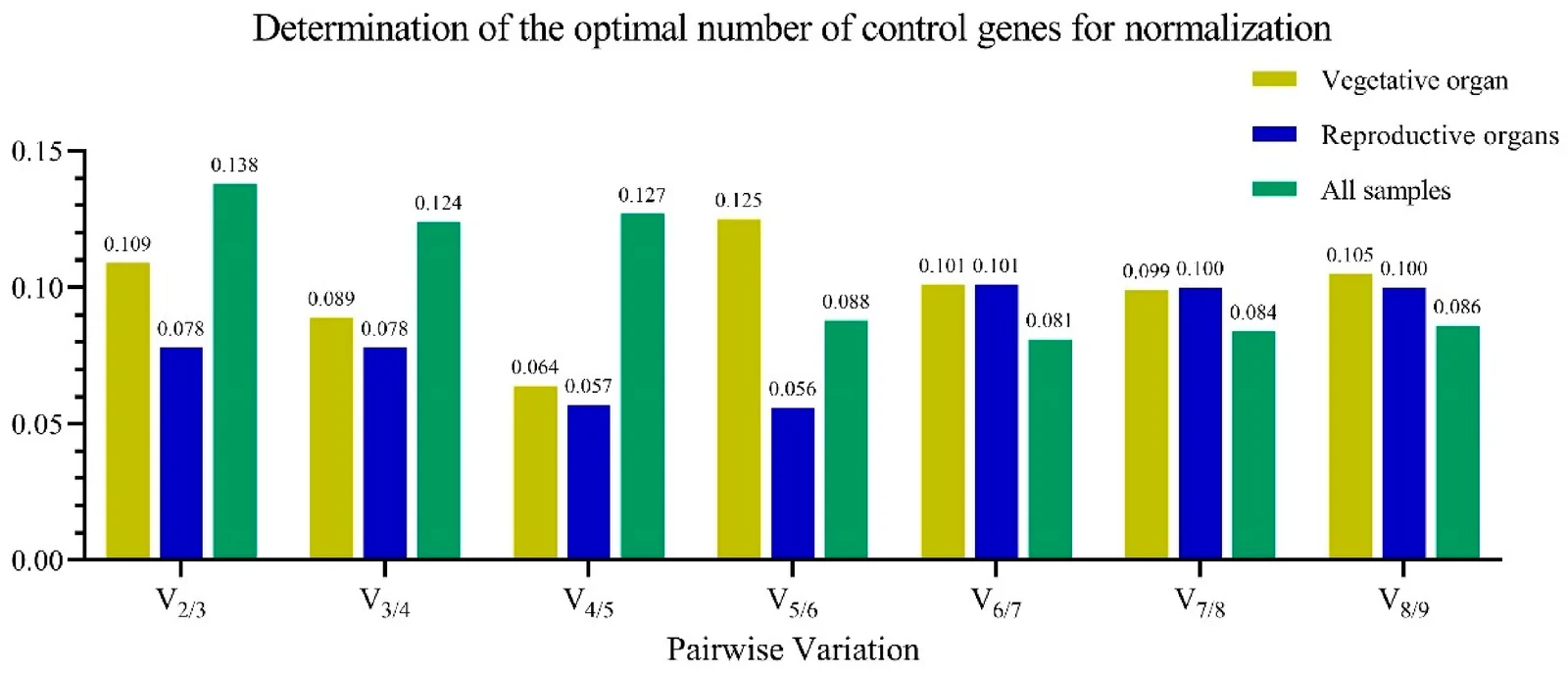
Pairwise variation analysis. Yellow, blue, and green bars correspond to the V_n/n+1_ in vegetative organs, reproductive organs, and all samples. The values above the bars represent the corresponding V_n/n+1_ numerical data.

The pairwise variation (V_n/n+1_) was used to determine the optimal number of reference genes. If V_n/n+1_ values are below 0.15, no additional reference genes are required [4]. In all subsets, including vegetative organs, reproductive organs, and all samples, the V_2/3_ values remained below 0.15, indicating that the two most stable reference genes are sufficient for normalization in future experiments (Figure 4). The bar charts of the three subsets suggest that V_4/5_ and V_5/6_ may serve as trend transition points. Both the reproductive and vegetative organ subsets exhibited a gradual decline before these points, followed by a sharp increase at V_5/6_ and V_6/7_, respectively. In contrast, the all-tested sample subset showed a marked decrease at V5/6. Within each subgroup, variations among V_6/7_, V_7/8_, and V_8/9_ were minimal, with changes remaining below 0.006.

#### 3.3.2. NormFinder Analysis

**Figure 5:**
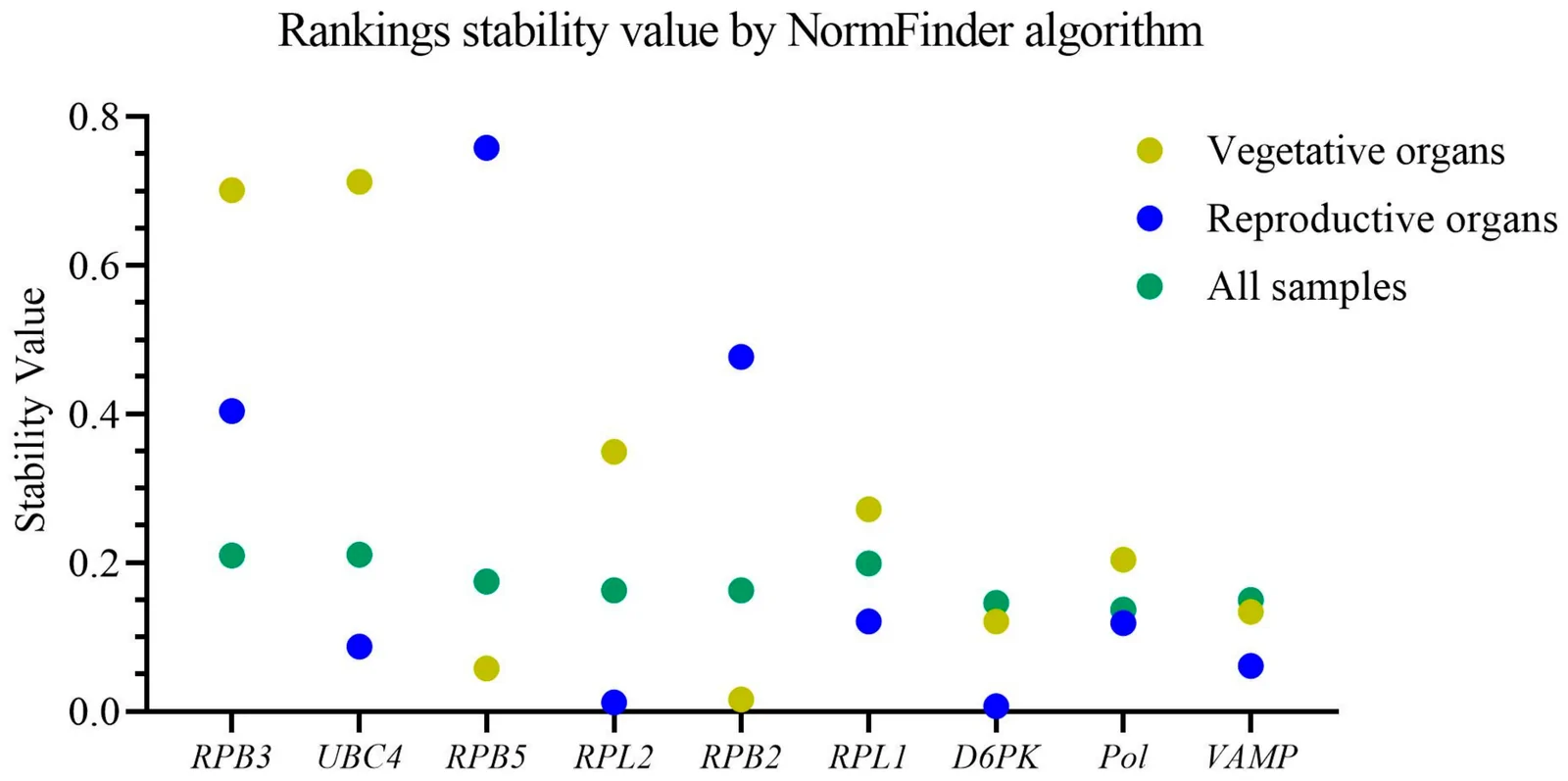
Rankings stability value by NormFinder algorithm. Yellow, blue, and green dots correspond to stability values in vegetative organs, reproductive organs, and all samples.

NormFinder analysis evaluated stability and ranked nine candidate reference genes (Figure 5). Lower stability values indicate greater suitability as reference genes. Candidate reference genes exhibited significant differences in stability values depending on the sample groups analyzed. When all experimental samples were included in the analysis, the stability values of all candidate genes (green dots) ranged from 0.137 (*Pol*) to 0.211 (*UBC4*), with an overall range of 0.74. In contrast, when analyzing vegetative organs and reproductive organs separately, the ranges increased to 0.696 and 0.751, respectively.

The ranking of candidate genes is based on their stability values across different sample groups (Table 4). For the combined analysis of all samples, the three most stable genes were *Pol*, *D6PK*, and *VAMP*, while *UBC4* was the least stable. In vegetative organs, the three most stable genes were *RPB2*, *D6PK*, and *VAMP*, with *UBC4* again identified as the least stable. For reproductive organs, the three most stable genes were *D6PK*, *RPL2*, and *VAMP*, whereas *RPB5* was the least stable. *Pol*, *D6PK*, and *VAMP* consistently ranked among the top performers across all three groups. *RPL2* and *RPB2* demonstrated superior stability in reproductive organs and vegetative organs, respectively. In contrast, *RPB3* was the poorest performer, consistently ranking among the bottom three.

#### 3.3.3. BestKeeper Analysis

BestKeeper ranked candidate reference genes according to the CV and SD. CV was expressed as the percentage of coefficient of variation, and SD represented the standard deviation of CP values. A lower CV indicated more stable gene expression, whereas a higher CV reflected greater instability. A larger SD denoted increased inconsistency in gene expression, with candidate genes having an SD > 1 deemed unsuitable and should be excluded.

**Figure 6:**
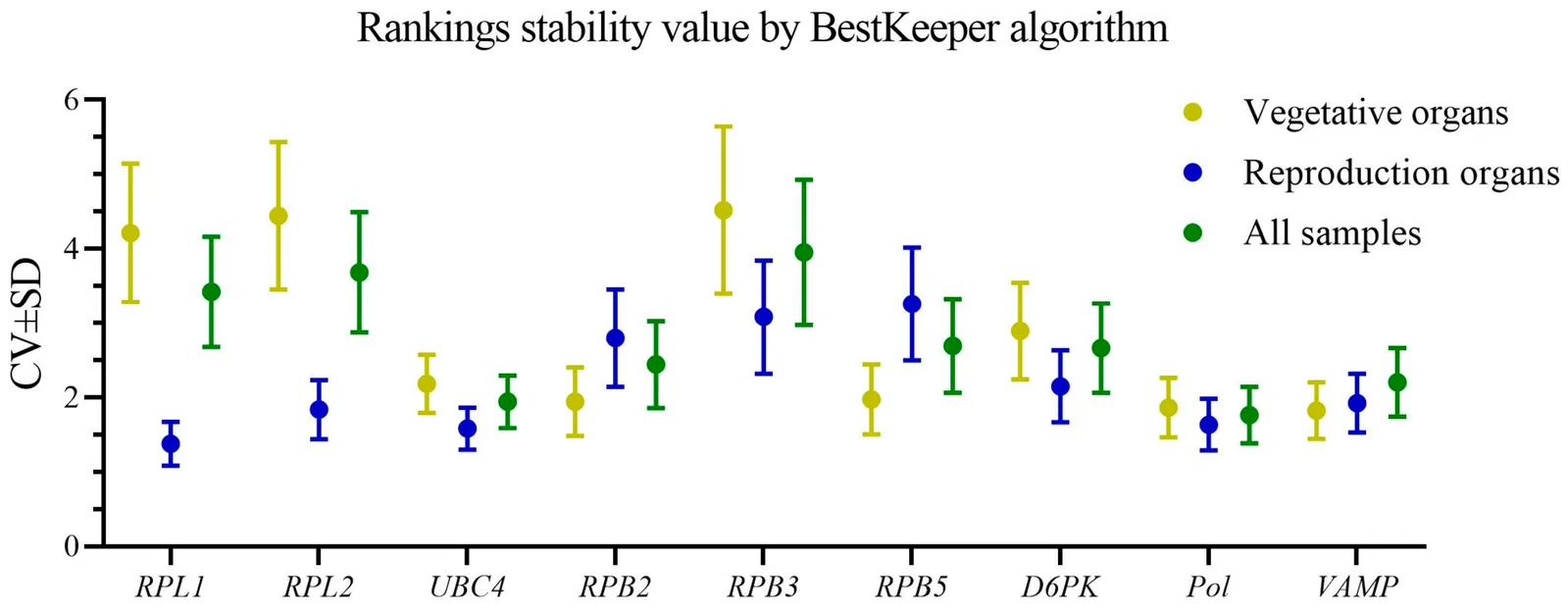
Rankings stability value by BestKeeper algorithm. Yellow, blue, and green patterns correspond to stability values in vegetative organs, reproductive organs, and all samples. Dots represented coefficient of variation (CV), and error bar showed standard deviation (SD).

Results demonstrated that *UBC4* and *Pol* performed excellently, consistently ranking among the top three across the three groups (Table 5). Specifically, *UBC4* exhibited the lowest SD in the reproductive organs (0.28) and all-samples group (0.35), while *Pol* showed the lowest CV in the all-samples group (1.76). *VAMP* displayed the lowest SD (0.38) and CV (1.82) in the vegetative organ group, indicating its suitability as a reference gene for vegetative organ studies. *RPL1* presented a unique performance profile. It was among the poorest-performing genes in the all-samples and the vegetative organ groups (Figure 6). But in the reproductive organ group, it showed the lowest CV (1.38) and the second-lowest SD (0.29). *RPB3* was the poorest-performing gene overall. Its SD and CV consistently ranked last in almost all groups; notably, it was the only gene with an SD > 1.

But in the correlation analysis results (Table 5), *UBC4* was not consistently identified as a stable reference gene. In the all-samples group and the vegetative organs group, it was the only candidate gene that showed no significant correlation with other genes (*p* > 0.05). However, in the reproductive organs group, its *p*-value was less than 0.01. It may only exhibit stable expression specifically in reproductive organs. Except for *UBC4*, all candidate reference genes showed strong and significant pairwise correlations.

#### 3.3.4. RefFinder Analysis

RefFinder integrates multiple computational programs, including the comparative Δ-Ct method. This method compared candidate reference genes by calculating the variation in Cp value (ΔCt) standard deviation (STDEV), where lower values indicate more stable expression levels [35]. The range of STDEV values among the top four genes in each group was less than 0.1 (Table 6). Specifically, *RPB2*, *RPL2*, and *D6PK* exhibited the lowest STDEV values in vegetative organs, reproductive organs, and all samples, respectively.

In addition, RefFinder integrates data from GeNorm, BestKeeper, and NormFinder to generate a geometric score (Table 7), where lower scores indicate greater suitability as reference genes. Specifically, *VAMP* exhibited the best performance in both the reproductive organ group and the all-samples group, *RPB2* ranked highest in the vegetative organ group, and *RPB3* remained the poorest-performing gene overall.

#### 3.3.5. Comprehensive Data Analysis

The top four rankings from four software tools were extracted for comprehensive analysis (Table 8). In the vegetative organ group and all-samples group, results from GeNorm and NormFinder were similar. BestKeeper ranks differed significantly from those of the other tools. *UBC4* appeared five times in the table; three of these occurrences were from BestKeeper, where it ranked either first or second. Overall, *VAMP* was the most widely applicable reference gene, as it was ranked first by at least one tool in all three groups. It was followed by *Pol*, which appeared nine times in the table. *RPB2* was likely the most suitable reference gene for vegetative organs, followed by *RPB5*. However, their performance was poor in the reproductive organ group. In the reproductive organ group, *VAMP*, *RPL2*, and *UBC4* exhibited excellent performance. In the all-samples group, *VAMP*, *Pol*, and *D6PK* performed well. They are the most suitable reference genes for all samples. Although *D6PK* ranked slightly higher than *Pol* in the RefFinder geometric mean, *Pol* exhibited superior cross-algorithm consistency. In contrast, *D6PK* was not ranked among the top four by BestKeeper in any sample group. Considering these results together, we selected *VAMP* and *Pol* as the optimal reference gene pair for *S. nigra* across diverse tissues.

#### 3.3.6. Validation of the Stability of Reference Genes

**Figure 7:**
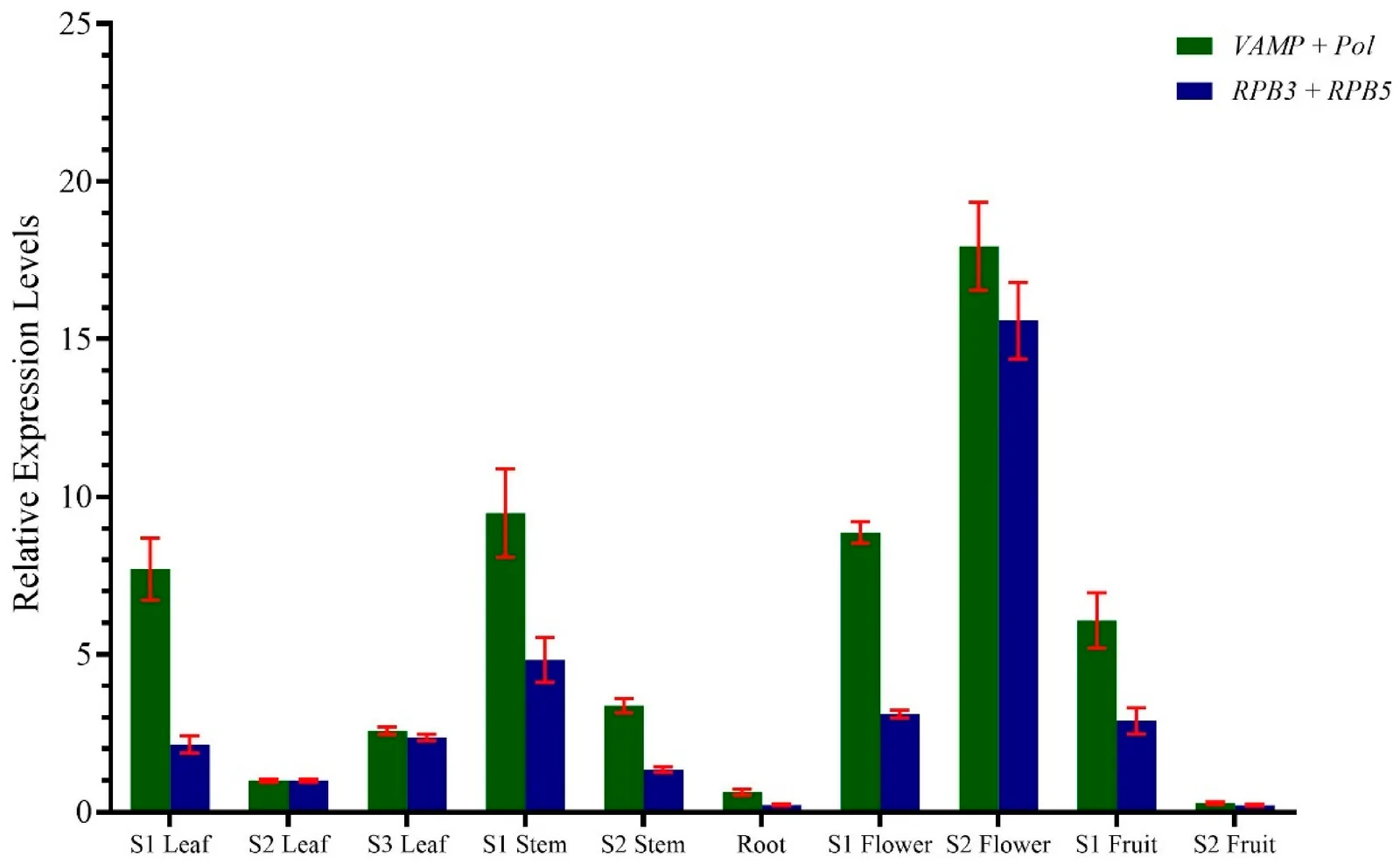
Relative expression levels of *SnAHCY* normalized by different reference genes. Based on results from four tools, *VAMP* + *Pol* and *RPB3* + *RPB5* were selected as the stable and unstable reference gene pairs, respectively. Error bars represent the standard deviation (SD) of three biological replicates (*n* = 3). These pairs were used to quantify the expression level of *SnAHCY*. Transcriptomic results indicated that *SnAHCY* exhibited extremely high expression levels in S1 stems, S1 flowers, and S2 flowers. The stable reference gene pair better reveals the true differences in gene expression across various tissues.

We selected *SnAHCY* as the target gene to demonstrate the impact of stable versus unstable reference gene combinations on data reliability. Using the relative expression method, we measured the expression levels of *SnAHCY* across various tissues with two reference gene pairs: the stable pair (*VAMP* + *Pol*) and the unstable pair (*RPB3* + *RPB5*) (Figure 7). As shown in the figure, the relative expression levels calculated by the two reference gene pairs differed significantly in some plant tissues. Furthermore, across all plant tissues, the relative expression levels calculated using the stable pair were consistently higher than those from the unstable pair. The normalization pattern obtained using the stable pair showed a high degree of consistency with the expression trend of *SnAHCY* derived from our transcriptomic data, whereas normalization using the unstable pair yielded a markedly different pattern. While this consistency suggests that stable pair normalization may better reflect the expected expression profile based on transcriptional data, it should be emphasized that this observation is correlative rather than proof of absolute accuracy.

## 4. Discussion

RT-qPCR is an important and widely used method for analyzing gene expression in plant molecular biology [36,37]. Its accuracy is affected by several experimental factors, including RNA integrity, reverse transcription efficiency, primer specificity, and amplification performance. Among these factors, the use of stable reference genes is particularly important for reliable normalization of gene expression data [38,39]. Although *S. nigra* has considerable ecological, nutritional, and medicinal value, molecular studies in this species remain limited, and validated reference genes have not been reported. In the present study, we systematically evaluated candidate reference genes in different tissues of *S. nigra* using multiple statistical algorithms. The results provide a reliable normalization framework for future RT-qPCR-based gene expression studies in this species.

Primer quality is an important prerequisite for reference gene validation. In RT-qPCR analysis, primers should show high specificity, produce a single amplification product, and exhibit appropriate amplification efficiency. In general, abnormal amplification efficiency may result from primer dimers, non-specific amplification, secondary structures in the template, unsuitable amplicon regions or sequence mismatches between primers and templates [40]. In this study, primers were designed to avoid obvious hairpin structures and primer dimers, but the amplification efficiency of several candidate genes still deviated from the expected range. This does not necessarily indicate that the primer design strategy was invalid. Instead, it may reflect the limitations of sequence information currently available for *S. nigra*. Because a high-quality annotated reference genome and complete full-length CDS dataset are still lacking for this species, primer design largely depends on available transcript sequences [41,42]. If these sequences contain assembly errors, incomplete transcript regions, alternative splicing variants, or single-nucleotide differences, primer-template matching and amplification efficiency may be affected. Tools such as Primer-BLAST can reduce the risk of nonspecific amplification and improve primer design reliability [43,44,45]. In addition, sequence-based models have also been developed to predict primer performance and amplification efficiency [46]. Nevertheless, for non-model species with limited genomic resources, primer performance should still be experimentally verified before reference gene stability analysis. Therefore, the observed variation in amplification efficiency in this study most likely reflects the combined effects of incomplete genomic resources and sequence-level uncertainty rather than a single primer design problem.

Previous studies have used the same types of analytical tools as those applied here to screen reference genes across different plant tissues and organs [47,48,49]. The present study was designed with two main aims. The first was to identify reference genes suitable for broad use across multiple tissue types of *S. nigra*. The second was to determine whether a single reference gene combination could be used for both vegetative and reproductive tissues. In some plant species, such as *Liriodendron chinense* and rice (*Oryza sativa*), the most stable reference genes differ between vegetative and reproductive organs [50,51]. This indicates that tissue type can strongly influence reference gene performance. In contrast, our results showed that the combination of *Pol* and *VAMP* was suitable for normalization across both vegetative and reproductive tissues of *S. nigra*. This broader applicability may be related to the initial selection of candidate genes based on transcriptome expression information. Screening candidates from transcriptome datasets can help exclude genes with obvious tissue-specific expression and increase the probability of identifying stable reference genes [52].

The stability rankings generated by geNorm, NormFinder and BestKeeper were not completely identical. This is expected because these programs use different statistical principles and computational procedures. geNorm focuses on pairwise expression variation among candidate genes; NormFinder estimates intra- and inter-group expression variation, and BestKeeper evaluates stability mainly using Ct-based statistical parameters. Therefore, different algorithms may rank the same candidate genes differently. However, this divergence is not a weakness of the analysis. Instead, it highlights the need for integrated evaluation. Relying on a single algorithm may lead to biased conclusions, whereas combining multiple tools can provide a more balanced and robust ranking [53]. In this study, RefFinder was used to integrate the outputs of different algorithms, allowing a comprehensive assessment of reference gene stability. This multi-algorithm strategy has also been used successfully in previous reference gene studies [50,51].

The expression stability of reference genes is not necessarily conserved across species or experimental conditions. Commonly used plant reference genes include *ACTIN*, *18S rRNA*, *CYP* and *UBC* [54,55,56]. However, these genes are not always suitable for all species or tissues. For example, Tu et al. reported that *ACTIN* showed the highest stability among candidate reference genes in *L. chinense* at different developmental stages [49], whereas *ACTIN* was not the best-performing gene in *S. nigra* in the present study. This difference further supports the view that reference genes must be validated for each specific experimental system. In general, ideal reference genes are often associated with basic cellular processes and maintain relatively constitutive expression, rather than being strongly inducible under specific conditions [57].

In this study, *VAMP* and *Pol* were identified as the most stable combination across different tissues of *S. nigra* (Table 8), indicating that these two genes are suitable for broad normalization in this species. The *VAMP* gene belongs to the *SNARE* (Soluble N-ethylmaleimide-sensitive factor attachment protein receptor) gene family [58]. Our literature search indicated that there are no published studies reporting the use of this gene as a reference gene in flowering plants. In *Arabidopsis*, *VAMP721* and *VAMP722* are key proteins involved in cell plate formation during cytokinesis and in exocytosis [59]. In the human body, *VAMPs* participate in multiple physiological processes, and their dysfunction facilitates the progression of various aging-related diseases [60]. However, some researchers have reported that the *VAMP* gene serves as an excellent reference gene in filamentous fungi [61]. It must be acknowledged that the evidence supporting *VAMP* as a suitable reference gene comes from studies conducted in filamentous fungi, which are evolutionarily distant from flowering plants. Gene regulatory mechanisms and expression profiles differ substantially between them. Domain analysis indicated that this *Pol* transcript contains a conserved RNA polymerase subunit 12-like domain, which may be present in multiple plant RNA polymerase complexes, and that *Pol* belongs to a class of genes encoding RNA polymerase [62]. In eukaryotes, these genes are structurally conserved and involved in ribosomal RNA synthesis [63,64]. The *18S rRNA* transcription is regulated by RNA Polymerase genes, which are commonly used as reference genes in plants and animals [65,66].

The three *RPB* genes showed distinct expression patterns in this study. *RPB2* and *RPB5* were stably expressed in vegetative tissues and ranked highly in several analyses, including geNorm. By contrast, *RPB3* showed the lowest stability and was consistently ranked near the bottom by different algorithms. *RPB* genes encode subunits of RNA Polymerase II and are directly involved in transcription and precursor RNA synthesis [67]. However, different *RPB* members may have distinct biological functions. For example, *RPB3* in *A. thaliana* is associated with stomatal development [68], and some animal *RPB* genes have been linked to gene silencing-related functions [69]. Therefore, functional divergence among *RPB* members may partly explain their different expression stability in *S. nigra*. Based on our results, *RPB2* and *RPB5* are recommended for studies focusing on vegetative tissues, whereas *RPB3* should be avoided as a normalization gene.

*RPL* genes encode ribosomal proteins and are generally associated with essential cellular processes [70]. Because of their relatively high expression and functional conservation, *RPL* genes have been used as reference genes in several plant species, including *Neolamarckia cadamba* [71], *Amaranthus tricolor* [72] and *Kentucky bluegrass* (*Poa pratensis*) [32]. In Kentucky bluegrass, *RPL* genes have been validated as suitable reference genes under heat stress conditions. However, the *RPL* homolog evaluated in *S. nigra* did not show consistently high stability across all tissue groups. It showed relatively large Ct variation and performed better only in reproductive tissues. This result again indicates that homologous genes may differ in expression stability among species and experimental contexts.

Although *D6PK* showed relatively stable expression across sample groups, it should be noted that D6PK has been reported to participate in blue light signal transduction and Polar auxin transport in *Arabidopsis*. Previous studies have linked *D6PK* to the regulation of Polar auxin transport [73]. This gene is directly involved in blue light signal transduction, and d6pk mutants display abnormal auxin transport [74]. Therefore, while D6PK is a suitable reference gene under the natural light conditions used in this study, caution is advised if it is to be employed in experiments involving artificial light manipulation or phototropic stress treatments.

*SnAHCY* encodes a member of the *S-adenosyl-L-homocysteine hydrolase* (*SAHH*) gene family, which is involved in plant development and floral morphogenesis [75]. Previous sequencing data indicated that this gene is highly expressed in young stems and leaves. In this study, *SnAHCY* was used to compare the normalization performance of stable and unstable reference genes. The results showed that using unsuitable reference genes led to a clear underestimation of *SnAHCY* expression levels (Figure 7). In some cases, the differences reached several-fold, which could seriously affect biological interpretation. This validation result confirms that inappropriate reference genes can introduce substantial errors into RT-qPCR analysis. Therefore, the use of stable reference gene combinations, such as *VAMP* + *Pol* for multiple tissues and *RPB2* + *RPB5* for vegetative tissues, is recommended for gene expression studies in *S. nigra*.

## 5. Conclusions

In this study, RT-qPCR, combined with geNorm, NormFinder, BestKeeper, and RefFinder, was used to evaluate the expression stability of candidate reference genes in different tissues of *S. nigra*. The comprehensive analysis identified *VAMP* and *Pol* as the most stable reference gene combination for multi-tissue normalization. For researchers conducting gene expression studies on purely vegetative tissues. *RPB2* and *RPB5* are recommended as the most reliable reference genes. These results provide a practical reference for accurate RT-qPCR normalization in *S. nigra* and will support future studies on gene expression, metabolite biosynthesis, and molecular breeding in elderberry.

## Figures and Tables

**Figure 1:**
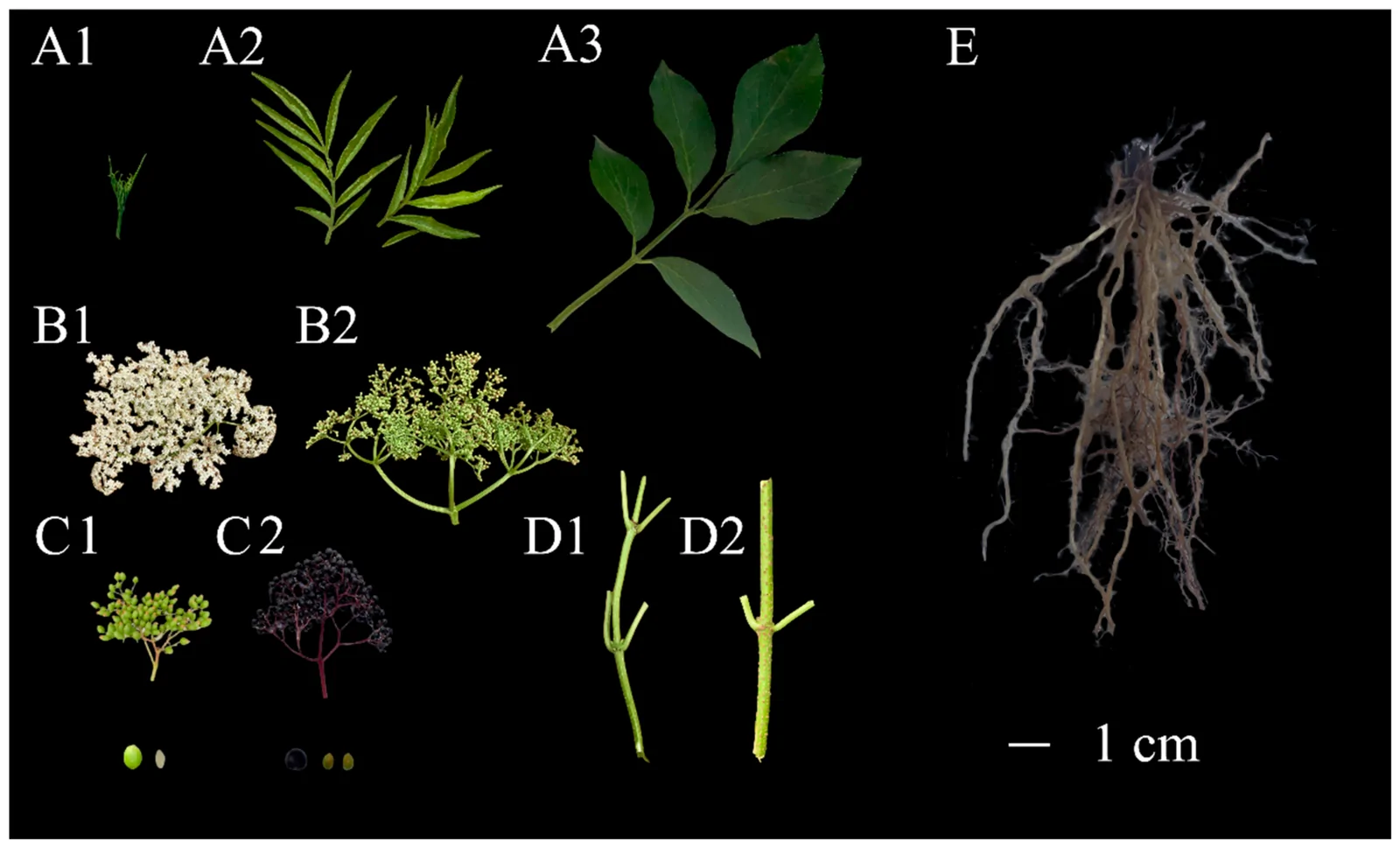
Test plant material sample collection diagram. The sample codes in Figure 1 correspond to those listed in Table 1.

**Table 2:** Primer sequences for RT-qPCR analysis.

Gene	Primer Sequences	Amplicon Size (bp)
*ACTIN*	Forward primer AGGAGGAAGGGAAGGTGAGT	144
	Reverse primer CCACCACCTCACAAACAACC	
*CYP1*	Forward primer TACAAGCACGCCCCCAAAAC	141
	Reverse primer TTCACCACCCCTTCCTGTCC	
*CYP2*	Forward primer TGGGATTCTGTGTTGGGCTT	121
	Reverse primer CGCTACGCTCGGGAAGAAAC	
*RPL1*	Forward primer ACATGAAAGTCAGGGCAGGC	112
	Reverse primer TTCCTGCGTCGATGGTCAAC	
*RPL2*	Forward primer AGGTGAATGGTGGAGATGCC	136
	Reverse primer TCGTACCCCTTGCCCTTAGT	
*UBC1*	Forward primer TACTCGGCCAGCTTGACAAC	118
	Reverse primer CCGCCACCAATTTCAGCATT	
*UBC3*	Forward primer GACGTTCTCTCGGGCTTGAT	182
	Reverse primer CTCGGCCACTGAAAGTTCGT	
*UBC4*	Forward primer TTCGAGCTGGAGGAGGTTGT	152
	Reverse primer GGGGTGTCGACGTGCTTTTT	
*RPB1*	Forward primer GGGGAGGACAATTGGAGCTT	96
	Reverse primer CCTTCCCCATCTCCACATCC	
*RPB2*	Forward primer CAGCGATTTGGAGTTGGCCT	115
	Reverse primer AGCAAGGCGTTCATTGTGGT	
*RPB3*	Forward primer TTGGGAGAGTGGAGTGGGTT	141
	Reverse primer ACCGGAATGCGTGACAAAGT	
*RPB5*	Forward primer GCTGACCAAAAACAGGCACC	119
	Reverse primer CCAAATACACCGGCAAGGAC	
*D6PK*	Forward primer GTGGCAAGAGCAGCATGTGT	137
	Reverse primer TCGGACCGCTTGGATTGCTT	
*Pol*	Forward primer TGAGCCAGTGAGTTACATCT	116
	Reverse primer TACGGGTTCTCTTCTTGTAC	
*VAMP*	Forward primer TTCTCCCCGCGATCTAGAAC	142
	Reverse primer GATCCAAGTTGAAGGAGCAT	

**Table 3:** Amplification efficiency and correlation coefficient of 14 candidate reference genes.

Candidate Reference Genes	Amplification Efficiencies/%	Correlation Coefficient/R^2^
*ACTIN*	124.7266	0.9932
*CYP1*	133.6661	0.9835
*CYP2*	137.6821	0.9831
*RPL1*	102.8797	0.9987
*RPL2*	103.3225	0.9986
*UBC1*	147.8726	0.9760
*UBC3*	123.4594	0.9881
*UBC4*	100.9786	0.9954
*RPB2*	85.07946	0.9860
*RPB3*	108.0886	0.9951
*RPB5*	101.1234	0.9987
*D6PK*	112.5656	0.9985
*Pol*	105.8628	0.9979
*VAMP*	103.1316	0.9989

*RPB1* was excluded from the efficiency analysis due to its dual melting peak.

**Table 4:** Expression stability ranking of 9 candidate reference genes by NormFinder.

Gene Name	Vegetative Organs Stability Value	Gene Name	Reproductive Organs Stability Value	Gene Name	All Samples Stability Value
*RPB2*	0.016	*D6PK*	0.007	*Pol*	0.137
*RPB5*	0.058	*RPL2*	0.012	*D6PK*	0.146
*D6PK*	0.121	*VAMP*	0.061	*VAMP*	0.15
*VAMP*	0.134	*UBC4*	0.087	*RPL2*	0.163
*Pol*	0.204	*Pol*	0.119	*RPB2*	0.163
*RPL1*	0.272	*RPL1*	0.121	*RPB5*	0.175
*RPL2*	0.349	*RPB3*	0.404	*RPL1*	0.199
*RPB3*	0.701	*RPB2*	0.477	*RPB3*	0.21
*UBC4*	0.712	*RPB5*	0.758	*UBC4*	0.211

**Table 5:** The BestKeeper analysis results. The first table displays the expression profile analysis of the candidate reference genes across different sample groups. The second table presents the correlation analysis among the candidate reference genes.

Gene Name	Vegetative Organ SD	Vegetative Organ CV	Gene Name	Reproductive Organ SD		Reproductive Organ CV		Gene Name		All Samples SD		All Samples CV
*VAMP*	0.38	1.82	*UBC4*	0.28		1.58		*UBC4*		0.35		1.94
*UBC4*	0.39	2.18	*RPL1*	0.29		1.38		*Pol*		0.38		1.76
*Pol*	0.40	1.86	*Pol*	0.35		1.63		*VAMP*		0.46		2.20
*RPB2*	0.46	1.94	*VAMP*	0.40		1.92		*RPB2*		0.58		2.44
*RPB5*	0.47	1.97	*RPL2*	0.40		1.84		*D6PK*		0.60		2.66
*D6PK*	0.65	2.89	*D6PK*	0.48		2.15		*RPB5*		0.63		2.69
*RPL1*	0.93	4.21	*RPB2*	0.66		2.80		*RPL1*		0.74		3.42
*RPL2*	0.99	4.44	*RPB5*	0.76		3.26		*RPL2*		0.81		3.68
*RPB3*	1.12	4.52	*RPB3*	0.76		3.08		*RPB3*		0.98		3.95
		**Gene Name**		***RPL1***	***RPL2***	***UBC4***	***RPB2***	***RPB3***	***RPB5***	***D6PK***	***Pol***	***VAMP***
Vegetative organ		Coeff. of corr. [R]		0.957	0.958	0.184	0.976	0.899	0.922	0.895	0.769	0.870
		*p*-value		0.001	0.001	0.464	0.001	0.001	0.001	0.001	0.001	0.001
Reproductive organ		Coeff. of corr. [R]		0.770	0.978	0.888	0.834	0.781	0.823	0.975	0.799	0.881
		*p*-value		0.003	0.001	0.001	0.001	0.003	0.001	0.001	0.002	0.001
All samples		Coeff. of corr. [R]		0.886	0.937	0.353	0.839	0.849	0.806	0.905	0.792	0.848
		*p*-value		0.001	0.001	0.055	0.001	0.001	0.001	0.001	0.001	0.001

**Table 6:** ΔCt-based stability rankings output by RefFinder.

Gene Name	Vegetative Organs Average of STDEV	Gene Name	Reproductive Organs Average of STDEV	Gene Name	All Samples Average of STDEV
*RPB2*	0.53	*RPL2*	0.44	*D6PK*	0.6
*RPB5*	0.57	*D6PK*	0.47	*VAMP*	0.61
*VAMP*	0.61	*VAMP*	0.49	*Pol*	0.63
*D6PK*	0.63	*UBC4*	0.5	*RPB2*	0.66
*Pol*	0.65	*Pol*	0.53	*RPL2*	0.67
*RPL1*	0.69	*RPL1*	0.55	*RPL1*	0.69
*RPL2*	0.73	*RPB2*	0.78	*RPB5*	0.74
*RPB3*	0.89	*RPB3*	0.8	*UBC4*	0.81
*UBC4*	0.92	*RPB5*	0.93	*RPB3*	0.87

**Table 7:** Stability ranking by RefFinder algorithm.

Gene Name	Vegetative Organs Geo-Mean of Ranking Values	Gene Name	Reproductive Organs Geo-Mean of Ranking Values	Gene Name	All Samples Geo-Mean of Ranking Values
*RPB2*	1.41	*VAMP*	2.45	*VAMP*	1.86
*RPB5*	2.11	*RPL2*	2.51	*D6PK*	1.97
*VAMP*	2.28	*UBC4*	2.63	*Pol*	2.21
*Pol*	4.16	*D6PK*	2.78	*RPB2*	3.72
*D6PK*	4.68	*Pol*	2.94	*UBC4*	4.76
*UBC4*	6.18	*RPL1*	4.56	*RPL2*	5.62
*RPL1*	6.24	*RPB2*	7	*RPL1*	6.24
*RPL2*	7.24	*RPB3*	8.24	*RPB5*	6.74
*RPB3*	8.24	*RPB5*	8.74	*RPB3*	9

**Table 8:** Comprehensive stability ranking of candidate reference genes across 4 algorithms.

Group	Ranks	GeNorm	NormFinder	BestKeeper	RefFinder
Vegetative organs	1	*RPB5*	*RPB2*	*VAMP*	*RPB2*
	2	*RPB2*	*RPB5*	*UBC4*	*RPB5*
	3	*D6PK*	*D6PK*	*Pol*	*VAMP*
	4	*Pol*	*VAMP*	*RPB2*	*Pol*
Reproductive organs	1	*VAMP*	*D6PK*	*UBC4*	*VAMP*
	2	*Pol*	*RPL2*	*RPL1*	*RPL2*
	3	*UBC4*	*VAMP*	*Pol*	*UBC4*
	4	*RPL2*	*UBC4*	*VAMP*	*D6PK*
All samples	1	*VAMP*	*Pol*	*UBC4*	*VAMP*
	2	*Pol*	*D6PK*	*Pol*	*D6PK*
	3	*D6PK*	*VAMP*	*VAMP*	*Pol*
	4	*RPB2*	*RPL2*	*RPB2*	*RPB2*

## Data Availability

The original contributions presented in this study are included in the article. Further inquiries can be directed to the corresponding author(s).

## Abbreviations

The following abbreviations are used in this manuscript:

*CYP*	Cyclophilin
*RPL*	50S ribosomal protein
*RPB*	RNA polymerase II subunits
*D6PK*	D6 Protein Kinase
*Pol*	RNA polymerase subunit 12
*VAMP*	Vesicle-associated membrane protein
*UBC*	Ubiquitin conjugating enzyme
*AHCY*	Adenosylhomocysteinase
